# Family Planning Experiences and Needs of Young Women Living With and Without HIV Accessing an Integrated HIV and SRH Intervention in Zimbabwe-An Exploratory Qualitative Study

**DOI:** 10.3389/fgwh.2022.781983

**Published:** 2022-05-19

**Authors:** Constancia V. Mavodza, Joanna Busza, Constance R. S. Mackworth-Young, Rangarirai Nyamwanza, Portia Nzombe, Ethel Dauya, Chido Dziva Chikwari, Mandikudza Tembo, Victoria Simms, Owen Mugurungi, Tsitsi Apollo, Bernard Madzima, Rashida A. Ferrand, Sarah Bernays

**Affiliations:** ^1^Biomedical Research and Training Institute, Harare, Zimbabwe; ^2^Department of Public Health, Environments and Society, Faculty of Public Health and Policy, London School of Hygiene and Tropical Medicine, London, United Kingdom; ^3^Department of Global Health and Development, Faculty of Public Health and Policy, London School of Hygiene and Tropical Medicine, London, United Kingdom; ^4^Clinical Research Department, Faculty of Infectious and Tropical Diseases, London School of Hygiene and Tropical Medicine, London, United Kingdom; ^5^MRC London School of Hygiene and Tropical Medicine, London, United Kingdom; ^6^Department of Infectious Disease Epidemiology, Faculty of Epidemiology and Population Health, London School of Hygiene and Tropical Medicine, London, United Kingdom; ^7^Ministry of Health and Child Care, HIV and TB Department, Harare, Zimbabwe; ^8^National AIDS Council, Harare, Zimbabwe; ^9^School of Public Health, University of Sydney, Sydney, NSW, Australia

**Keywords:** HIV, family planning, integrated service delivery, young people living with HIV, Zimbabwe

## Abstract

**Background:**

People living with HIV have higher unmet family planning needs compared to those without HIV. This is heightened for young people. However, the provision of family planning for young people within HIV programmes is uncommon. We investigated family planning uptake, acceptability of, and engagement with a service offering integrated HIV and sexual and reproductive health services for youth in a community-based setting in Zimbabwe.

**Methods:**

CHIEDZA, a community-based intervention offering integrated HIV and sexual and reproductive health services to young people aged 16–24 years, is being trialed in Zimbabwe. This exploratory qualitative study was nested within an ongoing study process evaluation. Data was collected between March-May 2021 with two sets of interviews conducted: I) twelve semi-structured interviews with young women living with HIV aged 17–25 years and II) fifteen interviews conducted with young women without HIV aged between 20 and 25 years who used a contraceptive method. A thematic analysis approach was used.

**Results:**

Before engaging with CHIEDZA, young women had experienced judgmental providers, on account of their age, and received misinformation about contraceptive use and inadequate information about ART-contraceptive interactions. These presented as barriers to uptake and engagement. Upon attending CHIEDZA, all the young women reported receiving non-judgmental care. For those living with HIV, they were able to access integrated HIV and family planning services that supported them having broader sexual and reproductive needs beyond their HIV diagnosis. The family planning preference of young women living with HIV included medium to long-acting contraceptives to minimize adherence challenges, and desired partner involvement in dual protection to prevent HIV transmission. CHIEDZA's ability to meet these preferences shaped uptake, acceptability, and engagement with integrated HIV and family services.

**Conclusions:**

Recommendations for an HIV and family planning integrated service for young people living with HIV include: offering a range of services (including method-mix contraceptives) to choose from; supporting their agency to engage with the services which are most acceptable to them; and providing trained, supportive, knowledgeable, and non-judgmental health providers who can provide accurate information and counsel. We recommend youth-friendly, differentiated, person-centered care that recognize the multiple and intersecting needs of young people living with HIV.

## Introduction

Two-thirds of people with HIV globally live in Sub-Saharan Africa (SSA) (1). In eastern and southern Africa, adolescent girls and young women (AGYW) aged 15–24 years old accounted for 26% of all new infections in 2018 (2). In Zimbabwe, HIV prevalence among adults in 2020 was estimated at 12.9% and the prevalence is higher among women (15.3%) compared to men (10.2%) (3). This gender disparity is even more pronounced among young people (20–24 years) with HIV prevalence nearly three times higher in young women (8.1%) than young men (2.1%) (4).

Numerous studies across diverse contexts have highlighted the family planning needs of people living with HIV, which include but are not limited to reducing HIV-positive births (5–8). Similar to HIV-negative women, women living with HIV (WLHIV) may wish to plan, avoid or delay pregnancies or limit family size (9). Historically, aside from condoms, few family planning methods were promoted or made easily accessible to WLHIV, undermining their health and well-being (10). Recent evidence indicates that they have lower contraceptive use and discontinue hormonal contraceptives more frequently than women without HIV (11, 12). One of the reasons for this is often unaddressed concerns about potential negative interactions between ART and hormonal contraceptives (13, 14). The few studies that have been conducted have shown limited data revealing that efavirenz-based ART may reduce the effectiveness of implants and combined oral contraceptives (15). Organizations and international bodies have created guidance materials to describe these interactions and provide a reference for service provision (16, 17). However, the limited data, clarity, and education on these interactions (15) adds additional complexity to the provision of HIV and family planning counseling, and further deters the provision of family planning within HIV programs (18–20).

WHO and other international bodies have made a case for integrating HIV and family planning services (21, 22). Previous studies show the acceptability of “*one-stop-shop”* approaches to improve both HIV and maternal health outcomes (23) and that such integration has resulted in increased contraceptive use among WLHIV (24–26).

There has been much less focus on the family planning needs of young people living with HIV (YPLHIV), and how approaches for older women may need to be adapted for younger women (27–29). Youth have high unmet need and already face considerable barriers to accessing family planning services (6, 30). These barriers include provider discrimination, lack of confidentiality, denial of young people's sexuality, parental consent mandates, and contraceptive use stigma (31). HIV is a chronic, already stigmatized condition that can pose additional barriers. Providers have reported low self-efficacy in their abilities to provide contraceptives to YPLHIV, due in part to poor knowledge about their SRH needs (32–34). Additionally, a review on the SRH needs of YPLHIV in low and middle-income countries found a paucity of clear policies to guide the provision of their SRH services (35). For young people who acquired HIV vertically, barriers include navigating the transitions from pediatric HIV programs where there are low/no exposure to sexual and reproductive health (SRH) services, to adult/youth HIV programs that may not recognize the need to introduce YPLHIV to SRH services (18, 19, 29, 36). A study in Uganda found that for these young people, there were no policies for youth-friendly transition clinics, and poor institutional and provider abilities to meet the SRH needs of YPLHIV (37). The evidence that exists suggests that there is limited consideration given to the SRH of YPLHIV and even lesser consideration for why their needs may differ from those without HIV (38). While there is a strong rationale for integrating HIV and family planning services, adaptations to address additional barriers to engagement and access faced by young women with HIV may be required.

We sought to understand young WLHIV's experiences accessing a community-based integrated service, exploring the acceptability, engagement, and uptake of HIV and family planning components. This includes comparing the experiences of young WLHIV and those without HIV to identify the influence of HIV status on youth's family planning needs and engagement in care.

## Methods

### Study Setting

CHIEDZA is a cluster randomized control trial investigating the impact of offering HIV testing and care with integrated SRH services to young people aged 16–24 years on population-level HIV outcomes in Zimbabwe (39). The trial is being conducted in three provinces: Harare, Bulawayo, and Mashonaland East, and this study was conducted as part of an embedded process evaluation of the intervention. The CHIEDZA intervention package has a ‘*one-stop approach'* (23) that was co-designed with young people (40) and provides HIV testing and counseling, HIV treatment and adherence support (to those who are HIV-positive), as well as family planning information and counseling, mixed-methods contraceptives, condoms, Sexually Transmitted Infections (STI) testing and management, menstrual health information and products, and general health counseling in a community-based setting. For HIV services, young people with newly or previously diagnosed HIV can choose to join the CHIEDZA HIV cohort by opting to receive ART through CHIEDZA. Those who choose to continue to access their ART from other settings and come to CHIEDZA for non-HIV SRH services are defined as not being in the HIV cohort. They are still able to access viral load monitoring and adherence support through CHIEDZA. All young women attending CHIEDZA are able and encouraged to access the range of family planning methods available. A team of trained health providers consisting of nurses, community health workers, youth workers, and counselors offer services at community centers. The team received training on not only their roles and responsibilities but also in providing confidential, youth-friendly services and engaging with young people. CHIEDZA seeks to further address access barriers by creating a space where young people can access services discretely through private health booths. They can engage in social activities, which can serve as both an incentive and an explanation for their attendance. Furthermore, the integrated nature of service provision makes it difficult to know what services a young person came to access (39, 40).

From April 2019 to July 2021, 1152 young women living with HIV engaged with CHIEDZA, of whom 1022 took up family planning services. All were receiving ART with 22.9% receiving ART through CHIEDZA (in CHIEDZA cohort), and the remainder in other care settings.

### Study Design

This study used an exploratory qualitative approach (41) to produce a detailed understanding of young WLHIV's experiences accessing integrated HIV and family planning services, and how these experiences have shaped uptake, acceptability, and engagement. This included examining through comparative analysis any differences in how WLHIV and those not living with HIV interact with and perceive integrated services.

### Data Collection and Analysis

We conducted two sets of semi-structured interviews between March to May 2021 in Harare, Bulawayo, and Mashonaland East. One set of interviews was with twelve young WLHIV, irrespective of their current contraceptive use. The other was with fifteen young women who had tested HIV negative within the 12 months before the interview and were using contraceptives. Efforts were made to have samples that represented the use of diverse family planning methods. Participants were identified using CHIEDZA providers' knowledge of clients' HIV status and their contraceptive use. The providers approached eligible clients accessing the service on scheduled interview days to gauge their interest in participating and referred those interested to the interviewers. Interviews were conducted by the three trained female researchers (CM, RN, and PN), who were unknown to the participants, and not involved in directly providing CHIEDZA services.

Data collection took place during a period of restrictions related to the COVID-19 pandemic. To minimize the need for extra travel for participants from their homes to the CHIEDZA sites, in-person interviews were conducted on the same day that clients were attending the CHIEDZA sites for services. While COVID-19 restrictions imposed the need to adopt a convenience sampling approach, it did mean we were able to conduct in-person interviews where privacy and uninterrupted discussion could be guaranteed. Relying on telephone interviews, by contrast, could have been hampered by limited reception and uncertainty around the privacy of the conversation and restricted to participants who owned phones (42). Only three eligible young WLHIV declined to participate due to time constraints and we were able to include considerable variation within the sample of young WLHIV (Table 1). All interviews were conducted using appropriate infection prevention control measures including the use of face-covering, social distancing, and conduct of the interviews outdoors.

**Table 1:** Female participant characteristics.

**HIV status**	**HIV positive (*n* = 12)**	**HIV negative (*n* = 15)**
Age	Three were aged 16–19 years, and nine were aged 20–25 years Median age (range): 22 (17–25 years)	All 15 were aged between 20 and 25 years Median age (range): 23 (20–25 years)
Marital status	Six were married, six were single/never married, two were divorced	11 married, one divorced, one separated, two in a relationship
Contraceptive Commodities	Three were accessing oral contraceptives and two depo-injectables at CHIEDZA	Three combined oral contraceptive pills (COC) seven Depo-Provera injectable
	Two had 3–5-year implants inserted before attending CHIEDZA	Seven Jadelle implants • 4/7 implants were not inserted at CHIEDZA • 3 of these 4 had come to CHIEDZA specifically for jadelle Removal
	Two were using only condoms One was pregnant Two were not using any contraceptive methods	4/15 had switched family planning methods
Dual protection	4/7 on contraceptives were also using condoms • $\frac{3}{4}$ had supportive partners • $\frac{1}{4}$ had just separated from their partner	
Mode of HIV infection	Five vertically acquired HIV, seven horizontally acquired HIV	n/a
ART care	Six were receiving their ART through CHIEDZA, Six were receiving their ART from other care settings	n/a
Parity	Seven had children, five did not have children 5/7 women with children indicated wanting to wait before having any more	All 15 women had children 2/15 used non-condom family planning methods pre-partum, 13/15 only started using non-condom family planning methods postpartum

Given the likely influence of personal contexts in shaping young WLHIV's experiences and needs, the potential vulnerabilities of this group, and the desire for the narratives to be shaped by the participants rather than the researchers, the researchers deliberately asked open-ended questions and then followed the direction of the participants' conversations. The researchers (RN, PN) conducting the first set of interviews had prior experiences interviewing people living with HIV and drew on their understanding of the potentially emotional nature of discussions around HIV, and their previous experiences to help participants feel comfortable. The second set of interviews also used open-ended questions and an unstructured style to examine clients' narratives and experiences of contraceptive access and use. All interviews were conducted in the participant's preferred language (English, Shona, or Ndebele), and covered the following topics: knowledge and use of contraceptive commodities; access to and uptake of HIV and/ or family planning services in/out of CHIEDZA; and partner involvement in HIV and/ or family planning decisions and care. The interviews were audio-recorded, lasted between 15 and 50 min, and on average lasted approximately 29 min. All the interviews were transcribed into English.

A thematic analytical approach that was both deductive and inductive was used due to the exploratory nature of the study (43). The two data sets were collected separately from each other and analyzed separately initially. These parallel analyses were then considered comparatively to explore HIV and family planning integration. For this study, the second set of interviews (young women living without HIV and using contraceptives) was used only for comparative analysis of prior care and family planning experiences. CM manually coded all transcripts. Inductive codes emerging from the data were iteratively developed and integrated with deductive codes that were developed *a priori* from the research questions. Analytical memo-ing (44) was used to elucidate the emerging themes. In this iterative process, CM, JB, SB, and CM-Y identified, discussed, and compared key themes. This analytical approach provided a guide for examining the perspectives of participants, highlighting similarities and differences, and generating unanticipated insights, that became relevant for nuanced understanding.

Anonymized quotes are used to exemplify the themes, with IDI#, HIV status, age, and contraceptive use. Two or more contraceptive methods placed next to each other indicate a change in methods. In Zimbabwe, Secure is the brand name for the progesterone-only contraceptive pill, Control is the brand name for combined oral contraceptive pills, Jadelle is a brand name for an implant and Depo is the shortened name referring to Depo Provera injectable.

### Ethical Approvals

Ethical approval was granted by the Medical Research Council of Zimbabwe (MRC/A/2266), the Biomedical Research and Training Institute Institutional Review Board (AP144/2018), and the London School of Hygiene and Tropical Medicine ethics committee (14652). All participants provided written informed consent. A waiver for the requirement of guardian consent was granted for those aged below 18 years.

## Results

A characteristic of this group was that some participants provided concise responses during their interviews. The young WLHIV were recruited not on account of their contraceptive use but on account of their status, such that for some of them, their accounts of family planning were limited which resulted in concise responses. Additionally, for some participants being interviewed about HIV and family planning was a novel experience and there was little familiarity in discussing these topics. The research approach was underpinned by a youth-friendliness and youth-led model such that interviews could be as short or as long as the youth participants wanted them to be, and the researchers were not aiming to change or adapt that.

Of the seven horizontally infected young women, six were infected by partners who did not inform them of their positive HIV status.

Three major topics were explored: (1) Prior (before CHIEDZA) family planning experiences (2) Family planning experiences of attending CHIEDZA, and (3) Family planning preferences.

### Prior Family Planning Experiences of All Young Women, Regardless of HIV Status

Prior experiences with judgmental providers, having received incorrect information on contraceptive eligibility and side effects, and exposure to mixed messages influenced young women's uptake, acceptability, and engagement with family planning services. This was particularly pronounced for those living with HIV compared to those without HIV.

### Judgemental Providers Discourage Continued Engagement

Prior to engaging with CHIEDZA, most participants regardless of HIV status had accessed HIV or family planning care in other care settings. They commonly experienced judgmental and negative attitudes related to their age or marital status. Across the sample, participants noted that provider attitudes discouraged consistent engagement with care.

“*When you want pills from the clinic, sometimes the staff at the clinic don't work many times and they work on their own time, and not working is normal. So, they can ignore you and make you feel like you are useless. So that's what they do sometimes, and they are ignorant and attend others”* (IDI03, HIV+, 23 years, Secure-Depo).

“*Imagine asking an old lady from the clinic about depo and its effects (laughs). The nurses will obviously look at you and start judging you. At the end of the day, you will not be open enough to tell her your problems.”* (IDI06, HIV+, 23 years, Depo).

“*You may be unfortunate to get scolded whilst in the queue at the clinics and that's a discouraging factor. Even at the pharmacies, you may be unfortunate to get served with a teller who had attitude and be scolded yet you want to buy contraceptives.”* (IDI11, HIV-, 21 years, Control-Depo).

### Inaccurate Knowledge Influences Contraceptive Use

Both participant groups accessed CHIEDZA with prior beliefs and information that influenced contraceptive use. Some participants believed that they should not use family planning before having children as this would make conception a challenge when they did eventually want to have children.

“*Well for now I am not taking any family planning because I have never had a baby, so I am not taking any. I was told that if you take family planning before having children it will be difficult to have children so that is why I do not use any family planning”* (IDI10, HIV+, 25 years, condoms).

“*I once heard that when you start using pills before having a baby, they will be depositing in your womb such that when you stop taking them and now want to have a child; it becomes difficult because the pills will still be in one's womb”* (IDI7, HIV-, 20 years, Jadelle).

“*I don't think it's a good idea to use for example Depo, yet you don't even have one child. Maybe when the time comes for you to have a child, you may take longer to conceive equivalent to the number of years your family planning method was. So, I think it becomes complicated for someone who hasn't had a child yet.”* (IDI10, HIV-, 25 years, Depo).

Other participants did not have adequate information to properly take their contraception. One young WLHIV detailed her past experiences. She was diagnosed with HIV a week before she gave birth to her first child. Appropriately, at 6 weeks after the birth of her child, she started taking progesterone-only oral contraceptives. However, she fell pregnant again when her first child was still 4 months old.

“*When l got my first child that's when I started to take [progesterone-only contraceptive] pills and I wasn't taking them regularly. I didn't have enough education on how they are taken. I don't know whether you would drink them every day or something. l thought you would take them when you wanted to have sex. That's what I used to do till I got pregnant again. So, I wasn't drinking them every day”* (IDI9, HIV+, 22 years, Secure-Jadelle-Control).

Across both groups, it was unusual to have accurate knowledge of contraception if unmarried. Participants associated knowledge and use of contraceptives with marriage. The limited knowledge was underpinned by normative judgment, whereby they avoided family planning to comply with expected moral standards.

“*I haven't thought much about family planning or using it because I am not yet married”* (IDI2, HIV+, 21 years, none).

“*Those who are not yet married but are using family planning methods are doing something which is not allowed”* (IDI10, HIV-, 25 years, Depo).

“*Before having my child, I didn't use anything to prevent myself from getting pregnant…Young girls must not take family planning pills because they won't be married.”* (IDI6, HIV-, 22 years, Control).

In these cases, these existing beliefs impacted demand for contraceptives when they did engage with CHIEDZA, with several opting not to seek family planning services.

### Circulating Mixed Messages: HIV and Contraceptive Interactions

Some participants **(*n* =** **6)** across both interview sets had heard mixed messages about drug interaction between ART and hormonal contraceptives. For young WLHIV, this impacted the contraceptive options and decision-making presented to them. One client had previously visited a health provider wanting one contraceptive method but was counseled to choose another due to drug interactions between ART and her initial choice.

“*When I went to some surgery in town, I told them that I am HIV positive, and I wanted to insert jadelle. I was told not to use jadelle. I was told that ARVs contain chemicals that may disrupt the effectiveness of the jadelle and that I could easily fall pregnant. So, they advised me to use Depo and that is what I have been using ever since.”* (IDI6, HIV+, 23 years, Depo).

“*I heard that a person living with HIV must not use Jadelle as a family planning method because the method overpowers ARVs hence making it difficult for an individual to suppress their virus.”* (IDI06, HIV-, 21 years, Control).

“*I heard that family planning pills are overpowered by ARVs so, when one takes them together it means one between the two pills will not work for their intended purpose”* (IDI6, HIV-, 23 years, Control-Depo).

Another incorrect message a participant had heard was that their viral load determined whether one should or should not use contraceptives.

“*It [contraception] works but it depends on how your viral load status is. You cannot have family planning when your viral load is high but when it's low you can do a family planning it depends on how your status is when you want to do family planning”* (IDI03, HIV+, 23 years, Secure-Depo).

When young WLHIV then came to CHIEDZA, they acted on previous information received and did not access the range of available contraceptives that they may have preferred.

### Family Planning Experiences of Young WLHIV Attending CHIEDZA

While the desire for youth-friendly services was universally expressed, some young WLHIV had not considered family planning options as being relevant to them because they were HIV positive, highlighting the critical need of incorporating wider services within HIV care programmes. The findings in this section drew on data from young WLHIV.

### Integrated HIV and Family Planning Service Provision for Widened Access

Integration of family planning with other services was considered convenient by all participants as it allowed for meeting multiple, simultaneous needs. Some participants came for every available service, and others combined specific services that they required. Some participants initially had heard about CHIEDZA only as offering menstrual pads and family planning. When they arrived for their first visit, they were surprised at the convenience of the range of services available to them. For young WLHIV, this included HIV services they had not immediately prioritized or considered.

“*I was told about [CHIEDZA] by someone who came here. Like when l was told it was a program related to pads and family planning only, but when I came here, I noticed that they are so many services offered…They took my viral load and I got counseling!”* (IDI4, HIV+, 22 years, Implant).

Another client initially came to CHIEDZA with reproductive issues after being informed by her friend that CHIEDZA would be able to help with her incessant bleeding problem. She subsequently was tested for HIV and STIs and tested HIV-positive.

“*She [her friend] said they check on the uterus and other things, so that's when l decided to go since I had been bleeding for some time… I sat on the bench, and I got inside and spoke to a lady, and she asked me why I'd come, my age, and all. So, I told her that I came to be checked in my uterus… she told me that we have other tests that we conduct, and she started to explain and then l agreed to be tested”* (IDI5, HIV+, 24 years, condoms).

The integrated approach enabled entry into either family planning or HIV care and then facilitated access to the other service. Some participants living with HIV who were not in the CHIEDZA cohort (i.e., were not diagnosed with HIV at CHIEDZA and continued to access ART elsewhere) similarly heard about CHIEDZA being a program providing family planning and menstrual hygiene products. Many came specifically for contraceptives and the highly acceptable counseling services. They also received viral load monitoring (with associated adherence support) at CHIEDZA. Their viral load samples were collected on the same day that they came for their 3-month Depo and oral contraceptives refill.

“*I first came here because I wanted contraceptives. When I got here, I was also given pads and that is when I got to know that they test as well…*. *the last time, I tested for viral load I went inside the nurse's booth, and she drew blood from my left arm, and she filled two test tubes. The nurse told me that she was taking blood samples so that they get to see the amount of virus in my blood. During the testing, the nurse also told me the importance of taking my medication as this will assist in having a low viral load”* (IDI6, HIV+, 23 years, Depo).

*The first time I went to CHIEDZA, I wanted to take family planning services and pads and I am glad that I came because I was offered all these services for free... Here at CHIEDZA, I come for family planning and my viral load* (IDI11, HIV+, 24 years, Control).

The availability of integrated services at CHIEDZA allowed these clients to customize the levels and content of their SRH and HIV care.

“*I was told that I could take my medication from CHIEDZA if I wanted to, but I felt that it was going to be a burden. You know being transferred from the facility to CHIEDZA then back to the facility again was going to be a problem. That is why I decided to stick to my facility…The nurses [public sector facility] have also been very friendly and nice. I remember when I started taking ARVs they even told me that I might face side effects. So, both CHIEDZA and the facility have been very supportive and friendly* (IDI11, HIV+, 24 years, Control).

Participants living with HIV reported that the integrated service package offered by CHIEDZA was able to support them in both their HIV and family planning needs, especially those related to adherence. Adopting a similar approach to supporting ART adherence, CHIEDZA providers also encouraged those who were sexually active to take contraception regularly.

“*CHIEDZA makes sure that I take my medication [ART] because at times when I think of it I stop and they call me and they counsel me so that I continue to take my medication. They tell me that 'a lot of people are taking their medication', so continue to drink your pills. They are the ones who encourage me to take my medication…CHIEDZA encourages family planning to avoid one having an unwanted pregnancy, so they have family planning, and they encourage to you to take pills or injection”* (IDI1, HIV+, 17 years, Depo).

The integration of HIV and family planning services was advantageous for these young women by encouraging both uptake and engagement with a wider constellation of services. If they returned for any given service, they would continue to experience the ease of taking up others, e.g., collecting pads and being encouraged to (re-) test for HIV. Uptake of services that clients may not have been specifically looking for or considered in the past was thus increased.

### Components of Youth-Friendliness to Meet HIV and Family Planning Needs

Participants felt that CHIEDZA demonstrated friendliness through the availability of supportive and non-judgmental providers. For young WLHIV, CHIEDZA providers perceived and interacted with them as more than just being HIV-positive. They felt that the providers acknowledged that they were sexually active with a range of SRH needs that deserved to be met, which in turn encouraged engagement and uptake of CHIEDZA services.

“*I come here for family planning and my viral load and so far, I have not encountered any problems in accessing these services. I love coming to CHIEDZA because the providers are very friendly, and they advise me a lot on a lot of things especially to do with sex as well as the use of protection.”* (IDI11, HIV+, 24 years, Control).

“*Yes, treatment is different here [CHIEDZA], you have all the time to ask some things and to understand everything”* (IDI4, HIV+, 22 years, Implant).

“*I prefer coming to CHIEDZA to seek family planning services because the nurses here are very friendly and they do not judge. Here at CHIEDZA, the services are very good, and I love the fact that the providers are young people who can relate to our experiences”* (IDI06, HIV+, 23 years, Depo).

‘Friendliness' was perceived as an accepting attitude toward their sexuality combined with the availability of the full range of SRH services that a young person may require. For young WLHIV, this particularly meant HIV being viewed as a manageable condition that need not subsume all their other needs and aspirations. ‘Friendliness' was also perceived to be the provider support young WLHIV received when they selected only services that they wanted, respecting their choice not to take up various options.

### Family Planning Preferences of Young WLHIV

Young WLHIV ‘s preference of family planning methods was influenced by their HIV status and the intersecting multiple “other” identities (marital status; with/without children).

### A Preference for Available Contraceptives That Do Not Require Daily Maintenance

For young WLHIV, adherence and side effects were already a challenge in relation to their ART. To potentially reduce pill burden and further adherence concerns with family planning, some of them sought contraceptives that did not require daily intake, which were free and readily available at CHIEDZA. Participants also reported switching from oral contraceptives to a medium or long-acting method because of side effects.

“*I use the implant… The issue of pills, I would forget, forgetting plus the control [combined oral contraceptive] pill would affect me. I would feel dizzy”* (IDI4, HIV+, 22 years, Implant).

“*I was still using Secure since I responded well to them. I recently got Depo because I sometimes used to forget to take the pills”* (IDI3, HIV+, 23 years, Secure-Depo).

“*I take Depo. Depo is better than pills because with Depo, with pills you forget but with Depo, you can last longer”* (IDI1, HIV+, 17 years, Depo).

For some, uptake of long-acting contraceptives was particularly associated with marital status. One participant who was not married but sexually active with an also HIV-positive partner used condoms only. She reported not taking contraceptives because she was not married yet, explaining that when she gets married, she would get the Jadelle implant because she would forget to take pills.

### Partner Influences Use of Condoms for Dual Protection

Most of the participants living with HIV articulated that regardless of their contraceptive use, condom use with their partners would offer dual protection against pregnancy, and HIV and STI transmission/reinfection. While condoms, contraceptives, and ART adherence services were readily available within CHIEDZA, their combined use by YPLHIV was shaped by relational dynamics. For young WLHIV who preferred to use dual protection, doing so was enabled by supportive partners. Some participants disclosed their HIV-positive status to partners who were accepting and supportive.

“*I just told him that I am positive, I don't know if you accept it or not. He said, ‘No problem we condomise'… he tells me to eat healthy food”* (IDI4, HIV+, 22 years, Implant).

“*I told him about it [disclosed status] and he has been supportive too as he encourages me to take my medication [ART] correctly all the time.”* (IDI6, HIV+, 23 years, Depo).

For those WLHIV who did not have supportive partners that encouraged dual protection and ART adherence, condom use was inconsistent:

“*At first, we used to wear condoms then, later on, we ended up not using protection since we are both positive and since no one is infecting the other one… currently we are using condoms… he refuses at times, but I force him to use”* (IDI8, HIV+, 18 years, condoms).

Across both samples, women in difficult or unsupportive relationships were less likely to use dual protection and used covert ways of preventing pregnancy. HIV-positive status amplified this. One client living with HIV switched from taking oral contraceptives to Depo injectable before her current pregnancy because her partner had wanted her to get pregnant and she wasn't ready. Similarly, another participant had a husband also living with HIV who did not adhere to his ART medication. He insisted on having another child with her when she was not ready.

“*My husband was now shouting at me. Since 2019 he wanted to have another child… so he would see it* [implant]. *Then he used to tell me to go and get it removed. He ended up shouting at me that ‘jadelle is meant for prostitutes. You put it for you not to get pregnant and do prostitution and have another sexual partner so that you don't get pregnant.' So, I went to get the jadelle removed and I never told him that l removed, and I came to CHIEDZA, and I collected control pills”* (IDI09; HIV+, 22 years, Jadelle-Control).

Her husband was unaware that she was now taking combined oral contraceptive pills covertly “*so now I take them every day at the same time as my [ART] medication”*.

## Discussion

In this study, we explored young WLHIV's experiences accessing HIV and family planning services in an integrated community-based intervention in Zimbabwe. This exploration included comparing experiences with those of young women without HIV. Our findings illustrate the SRH needs that are common amongst young women regardless of HIV status but by adopting a comparative approach we have also been able to identify the specific needs of young WLHIV. For young WLHIV, an integrative approach that includes providing both HIV and family planning services across their continuum of care was perceived to be acceptable and preferable to separate services. The importance of the provision of ‘youth-friendly' services to meet SRH needs has been extensively researched (45, 46). However, what ‘friendliness' entails is rarely examined in a nuanced way. For young WLHIV, being treated as more than just ‘HIV-positive', support for their agency, and integration of HIV care and family planning with flexibility around their care-seeking practices was important.

Our findings support those from other studies that have shown that supportive provider-client relationships are crucial for helping young people seek and adhere to ART and prevent unintended pregnancies through family planning care (47, 48). Therefore, in the context of HIV care, where individuals are seen repeatedly, there is an opportunity to develop such relationships. Discussions about family planning and fertility discussions could be layered onto such foundational relationships with providers within integrated HIV and SRH services (18–20, 49, 50). Additionally, provider support for women to select only the services that they wanted, including choosing not to take up certain options enables them to exercise agency. In our study, for example, some of these clients were accessing other care settings (sometimes for their ART), yet still chose to come to CHIEDZA for free family planning services and additional HIV support.

Our study's findings echo other studies that suggest that while HIV care programmes may adequately provide HIV treatment, they are insufficient in addressing the broader health needs of WLHIV with family planning (51) and HIV services are often separate and vertical (10). In addition, in many HIV programmes, the specific needs of young women within both adult and pediatric services are overlooked, particularly the SRH needs of those who are unmarried (29). Seeking family planning is a recognition of active sexual status (52), which for young people in Zimbabwe is often considered immoral behavior by parental figures and health practitioners in clinics. In CHIEDZA this is not only accepted but anticipated and accommodated for with a range of options, demonstrating the intent to provide an acceptable and convenient family planning option that is tailored to the individual needs of young women. The study demonstrates the importance of accepting, responsive and supportive services that for those living with HIV, acknowledges and views them as sexually active youth with a range of additional SRH needs.

Studies in eastern and southern Africa have noted the missed opportunities in providing family planning through HIV care and treatment programs (ART clinics) to reduce the unmet need for people living with HIV (53, 54). Some of our participants were vertically infected. In their HIV care trajectory, they had been exposed to pediatric HIV services which tend to deny their fertility or attempt to postpone their reproductive desires (55), or adult HIV services that may pass judgment as experienced by our participants (29). Zimbabwe, like many other countries in the region, has been making a concerted effort to integrated HIV and family planning services (56, 57). In cooperating family planning at ART clinics within a youth-friendly setting, could improve health outcomes for young WLHIV.

Like women without HIV, WLHIV had limited knowledge about family planning and contraceptive use, but in addition, had misguided presumptions about the effects of hormonal contraceptives. Concerns about drug-drug interactions and viral load being a determinant for eligibility to use contraceptives limited their perceived contraceptive options. Studies have shown that HIV providers have not always been confident in their knowledge to effectively provide quality family planning counseling for young WLHIV (18, 32–34, 49, 58). Mixed messages from providers and subsequent incorrect comprehension may result in WLHIV discontinuing either ART or contraception. Zimbabwe has successfully implemented and adopted at scale, the ZVANDIRI CATS programme to provide care and support for children, adolescents, and YPLHIV (59, 60). With training and support, there is potential to embed family planning education or information within this model as part of integrating HIV and family planning services for YPLHIV. Additionally, integration of HIV and family planning services must go beyond making commodities available to incorporate adequate training of providers, so they are well-equipped to address these issues and minimize the prevailing inadequate information about the use of family planning concurrently with ART.

The choice of family planning approaches was influenced by the need to minimize pill burden and daily maintenance. In the context of adherence, young WLHIV's preference for medium to long-acting contraceptive options because of a fear to forget taking pills daily may be revealing of adherence challenges with ART which also has to be taken daily. This association between adherence and preferred contraceptive method requires further investigation. Young WLHIV's preference for contraceptive options that did not require daily maintenance supports the potential of long-acting injectable ART (61) for improving adherence and virological suppression in young people, and the potential for combining delivery of long-acting ART with that of LARC. Importantly, the provision of choice of contraceptives enables YPLHIV to exercise more agency over their contraceptive choices.

Partners play a significant role in contraceptive decision-making and for young WLHIV. A study conducted with urban women of reproductive age in Zimbabwe reported that for WLHIV, male partners had more control in their intimate relationships and there was a greater association between positive HIV status postpartum, and male partners who ever refused to use a family planning method (62). Where feasible, providers can strongly encourage and support education which may result in improved HIV and family planning service delivery for young WLHIV (63).

In many instances, integration is usually examined only for a component of the cascade. For example, '*integrating HIV testing with family planning'*. A strength of this study was that by including women exposed to the whole HIV cascade (testing, care, and treatment, adherence support) and those accessing HIV care outside the CHIEDZA service, integration with family planning could be examined across this spectrum. Limitations are that the study had a small interview sample and only included CHIEDZA clients. Eligible women not accessing any family planning and /or HIV care were not included in this study and examining their access challenges may improve understanding and need for accessing and providing integrated HIV and family planning services. Further research is needed to examine some of the findings in this paper. Understanding research with young men living with HIV and the partners of young women living with HIV would provide a more comprehensive understanding as partners shape choices.

## Conclusion

Differentiated models of care that customize youth-friendliness to provide integrated HIV and family planning services that recognize the multiple and intersecting needs of young people are essential. The range of services offered (including method-mix contraceptives and LARCs), the ability of these young people to have agency over which services work for them; and the presence of supportive, knowledgeable, and non-judgmental health providers who can provide accurate information and counsel, could improve the uptake, acceptability, and engagement of HIV and family planning services by young WLHIV. Our findings highlight the need for further research co-designed with policymakers, implementors, and young people living with and without HIV to understand the provision and utilization of integrated HIV and family planning counseling and service provision, that reflect the diverse experiences and needs of young WLHIV.

## Data Availability Statement

The raw data supporting the conclusions of this article will be made available by the authors, without undue reservation.

## Ethics Statement

The studies involving human participants were reviewed and approved by Ethical approval was granted by the Medical Research Council of Zimbabwe (MRC/A/2266), the Biomedical Research and Training Institute Institutional Review Board (AP144/2018), and the London School of Hygiene and Tropical Medicine Ethics Committee (14652). All participants provided written informed consent. A waiver for the requirement of guardian consent was granted for those aged below 18 years. Written informed consent from the participants' legal guardian/next of kin was not required to participate in this study in accordance with the national legislation and the institutional requirements.

## Author Contributions

This paper was conceptualized by CM, JB, SB, and RF. RN and PN conducted the interviews, led by CM and CM-Y. CM led data analysis with support from SB and JB. CM led on writing this manuscript with SB and JB and support from CM-Y, RF, TA, VS, CD, MT, and ED who implement the CHIEDZA trial. CM conducts the process evaluation of the family planning intervention in the trial, with support and guidance from SB, JB, and CM-Y. RF is the principal investigator of the CHIEDZA trial. All authors contributed to the article and approved the submitted version.

## Funding

This study was supported by funding from the Wellcome Trust (206316_Z_17_Z) and the Fogarty International Center of the National Institutes of Health (D43 TW009539).

## Conflict of Interest

The authors declare that the research was conducted in the absence of any commercial or financial relationships that could be construed as a potential conflict of interest.

## Publisher's Note

All claims expressed in this article are solely those of the authors and do not necessarily represent those of their affiliated organizations, or those of the publisher, the editors and the reviewers. Any product that may be evaluated in this article, or claim that may be made by its manufacturer, is not guaranteed or endorsed by the publisher.
